# Impact of physician shift work implementation on mortality and length of stay in an emergency and critical care center: an interrupted time series analysis

**DOI:** 10.1186/s12913-026-14226-6

**Published:** 2026-02-18

**Authors:** Ryuta Nakae, Toru Takiguchi, Yutaka Igarashi, Toko Hirano, Takuro Hamaguchi, Naoki Tominaga, Kenta Shigeta, Shoji Yokobori

**Affiliations:** Department of Emergency and Critical Care Medicine, Nippon Medical School Hospital, 1-1-5, Sendagi, Bunkyo-ku, Tokyo, 113-8603 Japan

**Keywords:** Critical care, Emergency medical services, Health workforce, Personnel staffing and scheduling, Hospital mortality

## Abstract

**Background:**

Shift-based physician work systems are increasingly being introduced in emergency and critical care settings in Japan. However, evidence regarding their effects on patient outcomes—particularly mortality and intensive care unit (ICU) length of stay (LOS)—remains limited.

**Methods:**

This single-center retrospective observational study was conducted at the Emergency and Critical Care Center of Nippon Medical School Hospital in Tokyo, Japan. We evaluated whether implementation of a structured physician shift-based work system was associated with changes in in-hospital mortality and ICU LOS using an interrupted time series (ITS) design. All patients admitted between November 2017 and December 2024 were screened. Patients admitted during the COVID-19 pandemic period, those with cardiopulmonary arrest before or upon arrival, and elective surgical admissions were excluded. A structured two-shift physician work system was implemented in October 2023. The primary outcome was in-hospital mortality, and the secondary outcome was ICU LOS. Segmented ITS regression models were used to estimate level and slope changes before and after implementation. Additional models adjusted for monthly mean APACHE II scores and diagnostic category distributions to account for temporal changes in patient severity and case mix.

**Results:**

A total of 5,477 patients were included (pre-shift: 3,745; post-shift: 1,732). After adjustment, no clear change in mortality was identified at implementation (level change − 1.48%, 95% CI − 5.23 to 2.27) or in the subsequent trend (slope change + 0.05% per month, 95% CI − 0.26 to 0.36). LOS showed a pre-intervention downward trend (− 0.13 days per month, 95% CI − 0.35 to 0.09), followed by an immediate increase at implementation (+ 2.95 days, 95% CI 0.10 to 5.80). No meaningful change in post-intervention slope was observed (− 0.09 days per month, 95% CI − 0.33 to 0.15).

**Conclusions:**

Introduction of a structured physician shift system was not associated with an increase in in-hospital mortality. A transient increase in ICU length of stay was observed at the time of implementation, even after adjustment for monthly APACHE II scores and diagnostic category distributions. These findings describe temporal changes in selected clinical outcomes following system implementation and should be interpreted with caution given the potential for residual confounding and the limitations inherent to observational time series analyses.

**Supplementary Information:**

The online version contains supplementary material available at 10.1186/s12913-026-14226-6.

## Background

In recent years, physician work-hour reform has gained global attention as healthcare systems seek to reduce burnout, improve clinician well-being, and maintain safe and sustainable care delivery. These reforms typically include shift-based schedules, restrictions on duty hours, and limits on continuous work duration. Although widely discussed in postgraduate training and surgical disciplines, implementing such reforms in emergency medicine—where uninterrupted, 24/7 staffing is essential—presents unique operational challenges.

Prior studies have examined the effects of duty-hour restrictions, particularly among residents, on patient outcomes. In the United States, implementation of Accreditation Council for Graduate Medical Education (ACGME) duty-hour standards has produced mixed results [1]. While some investigations reported no significant change in patient outcomes following work-hour reform [2], others have demonstrated reductions in mortality [3] and serious medical errors [4]. Within emergency care settings, shift-based physician staffing has been associated with improvements in safety and operational performance [4, 5]. However, much of this literature has focused on process-based or surrogate outcomes rather than clinically meaningful endpoints such as mortality or hospital length of stay.

Despite increasing adoption of structured shift systems among emergency physicians, evidence regarding their impact on patient outcomes in high-acuity settings remains limited. Notably, few studies have evaluated shift-based work systems as a comprehensive intervention involving both attending physicians and trainees; most prior work has centered on resident duty-hour reforms alone. This gap is particularly relevant in emergency and critical care environments, where senior physician presence plays a critical role in decision-making and workflow.

To address this knowledge gap, we conducted an interrupted time-series (ITS) analysis to evaluate the effect of implementing a comprehensive shift-based physician work system—including both attending and resident physicians—on in-hospital mortality and intensive care unit (ICU) length of stay (LOS). By examining trends before and after the system’s introduction, this study aimed to clarify the clinical impact of a structured shift-based work model in an emergency and critical care center.

## Methods

### Study design and setting

This was a single-center retrospective observational study conducted at the Department of Emergency and Critical Care Medicine of Nippon Medical School Hospital (Tokyo, Japan), a tertiary-level emergency medical center that provides both emergency department (ED) and ICU services within a single integrated care system.

The center receives approximately 1,600–1,700 critically ill patients annually via direct emergency medical services transport or interfacility transfers; walk-in patients are not accepted. After initial evaluation and resuscitation in the ED area, patients are admitted to the ICU. The unit provides care for both adult and pediatric critically ill patients and includes a 48-bed ICU, which ranks among the largest ICUs in Japan. The medical staff comprises 16–18 board-certified emergency physicians and/or intensivists (including supervisors and attending specialists), 8–10 senior residents (postgraduate year 3–5), and 7–8 junior residents (postgraduate year 1–2). At least two board-certified emergency physicians or supervisors are present on-site at all times, including nights, ensuring continuous advanced critical care supervision.

### Study population

We included all patients admitted to the Emergency and Critical Care Center of Nippon Medical School Hospital between November 1, 2017, and December 31, 2024. Patients were excluded if they met any of the following criteria:

1. Admission during the COVID-19 pandemic period (April 1, 2020–May 7, 2023),
2. Cardiopulmonary arrest before or upon arrival, or
3. Admission for scheduled or elective surgical procedures (e.g., stoma closure after colostomy, cranioplasty following decompressive craniectomy, or removal of orthopedic fixation devices).

The COVID-19 pandemic period corresponds to the duration during which Japan implemented enhanced infection control and major healthcare system measures, prior to the downgrade of COVID-19 to a lower-risk, influenza-like infectious disease category under Japan’s Infectious Disease Control Act on May 8, 2023, as designated by the Ministry of Health, Labour and Welfare. This period was excluded because pandemic-related operational changes could introduce substantial confounding unrelated to the shift-work intervention.

### Data collection

Data were obtained from a hospital-based registry specifically maintained by the department for quality monitoring and research purposes. The registry captures demographic, physiological, diagnostic, and outcome data for all ICU admissions. For patients with multiple admissions during the study period, only the first admission was included in the analysis to avoid duplication.

Data entry was performed by a dedicated medical clerical assistant (Y.S.) who had completed formal training and passed the Japanese Medical Clerical Assistant Skills Certification Exam. Although not a national license, this certification reflects standardized training in medical documentation, clinical data abstraction, and hospital information management. All registry entries were routinely reviewed by supervising physicians (I.O. and R.N.) to ensure accuracy and completeness.

Primary diagnoses were categorized according to the diagnostic classification defined by the Japanese national reimbursement system for emergency medical care, which is used to determine the emergency medical admission fee. Classification categories included cerebrovascular disease, respiratory failure, heart failure, poisoning, gastrointestinal bleeding, metabolic disorders, burn injuries, acute abdomen, trauma, and others. In-hospital outcomes were followed until discharge or death during hospitalization.

### Physician work style reform

In Japan, physician work style reform has been promoted by the Ministry of Health, Labour and Welfare to address longstanding concerns regarding excessive working hours and physician burnout. Under this national framework, the “Category A” standard serves as the baseline requirement for hospital-based physicians, limiting overtime to ≤ 960 h per year (approximately 80 h per month) and capping continuous working hours at approximately 28 h per shift. A minimum rest interval of 9 h between consecutive shifts is also recommended. In addition, healthcare institutions must implement physician health protection measures, including periodic medical evaluations, objective monitoring of working hours, and mandatory occupational health consultations for physicians who exceed designated thresholds. These regulations are intended to support physician well-being and maintain safe, sustainable healthcare delivery.

In alignment with this national initiative, our institution introduced a structured two-shift physician work system in October 2023. Under this system, all attending physicians and residents transitioned from a traditional extended-duty schedule to a model comprising two fixed daily shifts with mandatory rest intervals and standardized handovers. The daytime shift was 8 h (08:30–16:30), and the nighttime shift was 16 h (16:30–08:30). Physicians were scheduled for approximately 150–160 working hours per month, including 4–5 night shifts, with total overtime limited to ≤ 80 h per month, and with physician working hours objectively monitored using an electronic time-stamping system mandated by the institution. This reform represented a comprehensive restructuring of physician scheduling across all training levels and constituted the intervention evaluated in this study. For analytic purposes, admissions before October 2023 were classified as the pre-shift group, and those in October 2023 or later were classified as the post-shift group, corresponding to the implementation of the structured shift-based work system. Additionally, nursing staff schedules remained unchanged throughout the study period and continued to follow a two-shift system, with no major structural or staffing changes.

### Outcomes and definitions

The primary outcome was in-hospital mortality, defined as death from any cause occurring during the index hospitalization. The secondary outcome was LOS, defined as the number of calendar days from ICU admission to ICU discharge or transfer to another hospital department or external facility. LOS was calculated irrespective of survival; patients who died during hospitalization were retained in the analysis. For patients transferred to other institutions, LOS was counted up to the date of transfer. All outcome data were obtained from the institutional registry.

### Statistical analyses

Baseline patient characteristics were compared between the pre- and post-intervention groups to evaluate potential differences in case mix. Variables extracted included age, sex, vital signs at admission (Glasgow Coma Scale [GCS] score, blood pressure, heart rate, respiratory rate, and body temperature), illness severity measured using the Acute Physiology and Chronic Health Evaluation II (APACHE II) score [6], primary diagnostic category based on the Japanese emergency critical care reimbursement system, and treatment variables including mechanical ventilation, continuous renal replacement therapy (CRRT), and surgery. Continuous variables were summarized using mean (standard deviation [SD]) when approximately normally distributed and median (interquartile range [IQR]) when skewed. Categorical variables were reported as numbers and percentages. Between-group comparisons used the *χ²* test for categorical variables and the Student’s *t*-test or Mann–Whitney *U* test for continuous variables, as appropriate.

To evaluate the effect of implementing the shift-based physician work system, we performed segmented ITS analyses using ordinary least-squares regression. Monthly aggregated data were generated for all eligible admissions between November 2017 and December 2024, with October 2023 designated as the intervention point.

The ITS model included three terms:

1. Time, a continuous monthly variable representing the underlying secular trend before the intervention;
2. Intervention, a binary indicator coded 0 before and 1 after October 2023, estimating the immediate level change;
3. Post-intervention time, defined as (Time − intervention month), representing the change in slope following the intervention.

This model structure allows estimation of both abrupt level changes and modifications in temporal trends. Autocorrelation of residuals was assessed using the Durbin–Watson statistic, which did not indicate meaningful first-order autocorrelation; thus, ordinary least-squares estimation was retained. Model assumptions—including linearity, homoscedasticity, and approximate normality of residuals—were evaluated using residual plots and were deemed acceptable for segmented ITS analysis. Seasonality was examined and did not materially affect model fit. Sensitivity analyses were performed by additionally adjusting ITS models for monthly mean APACHE II scores and distributions of diagnostic categories to address potential case-mix differences. In addition, to further characterize temporal changes in illness severity distribution, we examined the proportion of patients with APACHE II score ≥ 20 and assessed overall trends using Kendall’s rank correlation.

All analyses were conducted using IBM SPSS Statistics version 25.0 (IBM Corp., Armonk, NY, USA). Effect estimates are reported with 95% confidence intervals, and statistical inference was based on two-sided *p* values < 0.05.

## Results

### Characteristics of study subjects

A total of 5,477 patients were included in the analysis: 3,745 in the pre-shift group and 1,732 in the post-shift group. Baseline characteristics are summarized in Table 1. The median age of the cohort was 67 years (IQR 45–79), and 62.2% were male, with no notable differences in age or sex distribution between groups. Vital signs at admission—including GCS score, heart rate, and body temperature—were generally comparable. Systolic and diastolic blood pressures were modestly lower in the post-shift group (136 ± 37 mmHg vs. 139 ± 36 mmHg, *p* = 0.004; 78 ± 30 mmHg vs. 79 ± 22 mmHg, *p* = 0.01). Respiratory rate was slightly higher in the post-shift group (25 ± 9 vs. 24 ± 8 breaths/min, *p* < 0.001).

**Table 1:** Characteristics of the study population

Characteristic	Total (*n* = 5,477)		Pre-shift group (*n* = 3,745)		Post-shift group (*n* = 1,732)		*p*
**Demographics**							
Age, median (IQR), years	67 (45–79)		67 (45–79)		67 (45–79)		0.52
Male, *n* (%)	3,408 (62.2)		2,350 (62.8)		1,058 (61.1)		0.24
**Vital signs at admission**							
GCS score, median (IQR)	14 (10–15)		14 (10–15)		14 (10–15)		0.72
Systolic blood pressure, mean ± SD, mmHg	138 ± 37		139 ± 36		136 ± 37		0.004
Diastolic blood pressure, mean ± SD, mmHg	79 ± 25		79 ± 22		78 ± 30		0.01
Heart rate, mean ± SD, /min	99 ± 28		99 ± 28		99 ± 28		0.22
Respiratory rate, mean ± SD, /min	24 ± 8		24 ± 8		25 ± 9		< 0.001
Body temperature, mean ± SD, ℃	36.8 ± 1.4		36.8 ± 1.4		36.8 ± 1.5		0.92
**Clinical score**							
APACHE II score, median (IQR)	13 (8–19)		12 (8–18)		14 (9–19)		< 0.001
**Primary diagnosis**							< 0.001
Cerebrovascular disease, *n* (%)	942 (17.2)		654 (17.5)		288 (16.6)		
Respiratory failure, *n* (%)	638 (11.6)		403 (10.8)		235 (13.6)		
Heart failure, *n* (%)	395 (7.2)		245 (6.5)		150 (8.7)		
Intoxication, *n* (%)	406 (7.4)		256 (6.8)		150 (8.7)		
Gastrointestinal bleeding, *n* (%)	669 (12.2)		467 (12.5)		202 (11.7)		
Metabolic disorder, *n* (%)	317 (5.8)		189 (5.0)		128 (7.4)		
Burn, *n* (%)	66 (1.2)		46 (1.2)		20 (1.2)		
Acute abdomen, *n* (%)	630 (11.5)		438 (11.7)		192 (11.1)		
Trauma, *n* (%)	1,071 (19.6)		767 (20.5)		304 (17.6)		
Others, *n* (%)	343 (6.3)		280 (7.5)		63 (3.6)		
**Treatments**							
Mechanical ventilation, *n* (%)	1,617 (29.5)		1,157 (30.9)		460 (26.6)		0.001
CRRT, *n* (%)	523 (9.5)		334 (8.9)		189 (10.9)		0.02
Surgery, *n* (%)	1,251 (22.8)		894 (23.9)		357 (20.6)		0.007

Pre-shift group: Patients admitted before the implementation of the shift-based work system (November 2017 – September 2023)

Post-shift group: Patients admitted after the implementation of the shift-based work system (October 2023 – December 2024)

Illness severity differed between groups: the median APACHE II score was higher in the post-shift period (14 [IQR 9–19] vs. 12 [IQR 8–18], *p* < 0.001). The distribution of primary diagnostic categories also varied (*p* < 0.001): respiratory failure, heart failure, intoxication, and metabolic disorders were more frequent in the post-shift group, whereas trauma and “other” conditions were more common pre-shift. Treatment profiles showed group differences as well. Mechanical ventilation was used more often in the post-shift group (30.9% vs. 26.6%, *p* = 0.001), CRRT use differed between groups (8.9% vs. 10.9%, *p* = 0.02), and surgical intervention occurred in 23.9% vs. 20.6% of patients (*p* = 0.007).

There were no missing data for any study variables. Compliance with the nationally mandated overtime limit of ≤ 960 h/year was 100% throughout the study period. Post-implementation physician working hours, stratified by postgraduate year, are summarized in Supplementary Table 1. The number of ICU nursing staff remained stable across periods, with no significant difference (148 [IQR 140–150] vs. 150 [IQR 145–150], *p* = 0.15).

### Study outcomes

In-hospital mortality was 6.9% in the pre-shift group (260/3,745) and 7.8% in the post-shift group (135/1,732), with no significant difference between groups (*p* = 0.26). The median LOS was shorter in the post-shift group compared with the pre-shift group (5 [IQR 2–13] vs. 7 [IQR 3–16] days; *p* < 0.001) (Table 2).

**Table 2:** Clinical outcomes in the pre- and post-shift groups

	Total (*n* = 5,477)		Pre-shift group (*n* = 3,745)		Post-shift group (*n* = 1,732)		*p*
**Primary outcome**							
Mortality, *n* (%)	395 (7.2)		260 (6.9)		135 (7.8)		0.26
**Secondary outcome**							
Length of stay, median (IQR), days	6 (2–15)		7 (3–16)		5 (2–13)		< 0.001

Pre-shift group: Patients admitted before the implementation of the shift-based work system (November 2017 – September 2023)

Post-shift group: Patients admitted after the implementation of the shift-based work system (October 2023 – December 2024)

### Interrupted time series (ITS) analysis

**Fig. 1:**
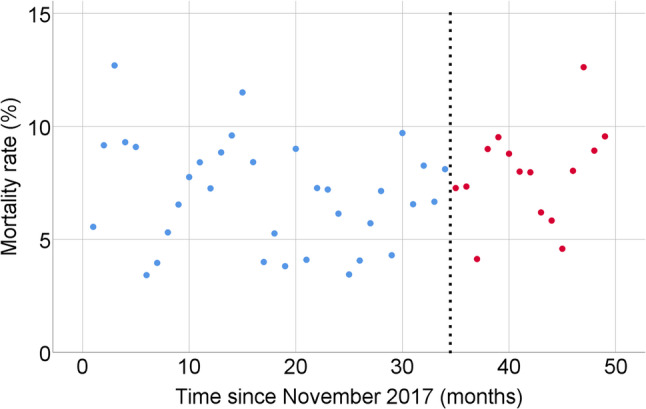
Interrupted time series analysis of monthly in-hospital mortality before and after implementation of the shift-based physician work system. Each point represents the monthly in-hospital mortality rate (%). Blue dots indicate the pre-intervention period and red dots indicate the post-intervention period. The vertical dashed line denotes the time of intervention (October 2023)

**Fig. 2:**
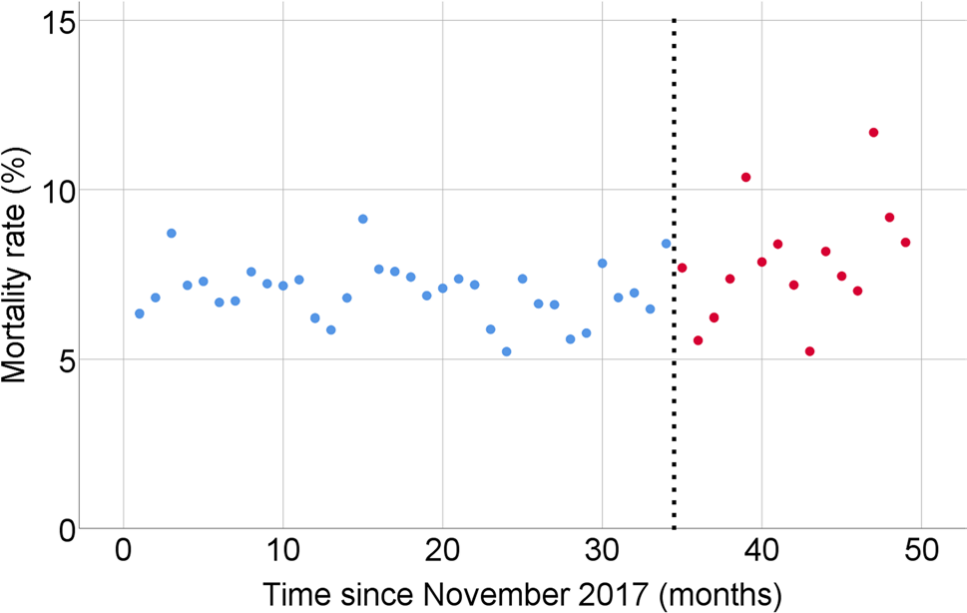
Interrupted time series analysis of monthly in-hospital mortality after adjustment for mean APACHE II scores and diagnostic category distributions. Each point represents the adjusted monthly in-hospital mortality rate (%). Blue dots indicate the pre-intervention period and red dots indicate the post-intervention period. The vertical dashed line denotes the time of intervention (October 2023)

ITS analysis for monthly in-hospital mortality showed no significant pre-intervention trend in the unadjusted model (B = − 0.05, 95% CI − 0.13 to 0.03; *p* = 0.26). The estimated level change at the intervention was + 0.43% (95% CI − 2.42 to 3.28; *p* = 0.77), and the post-intervention slope change was + 0.19% per month (95% CI − 0.08 to 0.46; *p* = 0.18) (Table 3; Fig. 1). In the adjusted model incorporating monthly mean APACHE II scores and diagnostic category distributions, the pre-intervention trend was B = 0.00 (95% CI − 0.01 to 0.02; *p* = 0.40), the level change at the intervention was − 1.48% (95% CI − 5.23 to 2.27; *p* = 0.44), and the post-intervention slope change was + 0.05% per month (95% CI − 0.26 to 0.36; *p* = 0.75) (Table 3; Fig. 2).

**Table 3:** Interrupted time series analysis for in-hospital mortality, before and after adjustment

Variable	Coefficient (B)	95% CI	*p*	Coefficient (B) (adjusted)	95% CI (adjusted)	*p* (adjusted)
Time	−0.05	−0.13–0.03	0.26	0.00	−0.01–0.02	0.40
Intervention	+ 0.43	−2.42–3.28	0.77	−1.48	−5.23–2.27	0.44
Post-intervention time	+ 0.19	−0.08–0.46	0.18	+ 0.05	−0.26–0.36	0.75

**Fig. 3:**
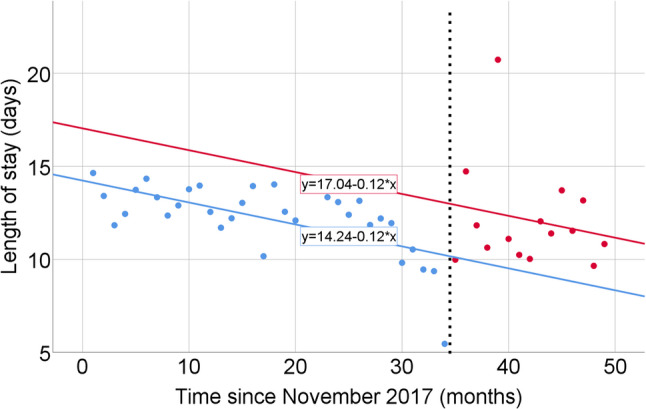
Interrupted time series analysis of monthly mean intensive care unit length of stay (LOS) before and after implementation of the shift-based physician work system. Each point represents the monthly mean LOS (days). Blue dots indicate the pre-intervention period and red dots indicate the post-intervention period. The vertical dashed line denotes the time of intervention (October 2023)

**Fig. 4:**
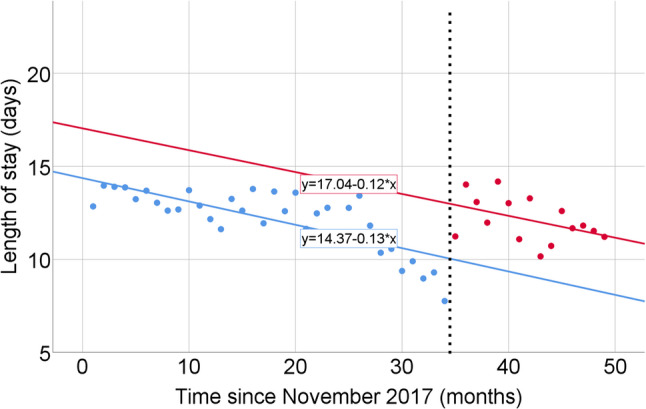
Interrupted time series analysis of monthly mean intensive care unit length of stay (LOS) after adjustment for mean APACHE II scores and diagnostic category distributions. Each point represents the adjusted monthly mean LOS (days). Blue dots indicate the pre-intervention period and red dots indicate the post-intervention period. The vertical dashed line denotes the time of intervention (October 2023)

For LOS, the unadjusted model showed a decreasing pre-intervention trend (B = − 0.12 days per month, 95% CI − 0.19 to − 0.05; *p* = 0.001). The estimated level change at the intervention was + 2.82 days (95% CI 0.40 to 5.24; *p* = 0.03), and the post-intervention slope change was 0.00 days per month (95% CI − 0.24 to 0.24; *p* = 1.00) (Table 4; Fig. 3). In the adjusted analysis, the pre-intervention trend was B = − 0.13 days per month (95% CI − 0.35 to 0.09; *p* = 0.25), the level change at the intervention was + 2.95 days (95% CI 0.10 to 5.80; *p* = 0.043), and the post-intervention slope change was − 0.09 days per month (95% CI − 0.33 to 0.15; *p* = 0.45) (Table 4; Fig. 4). To further assess temporal changes in illness severity, a trend analysis of the monthly proportion of patients with APACHE II score ≥ 20 was performed using Kendall’s rank correlation. No significant temporal trend was observed across the study period (*τ* = 0.15, *p* = 0.13), indicating that the burden of severely ill patients remained stable over time (Supplementary Table 2, Fig. 1).

**Table 4:** Interrupted time series analysis for length of stay, before and after adjustment

Variable	Coefficient (B)	95% CI	*p*	Coefficient (B) (adjusted)	95% CI (adjusted)	*p* (adjusted)
Time	−0.12	−0.19–−0.05	0.001	−0.13	−0.35–0.09	0.25
Intervention	+ 2.82	0.40–5.24	0.03	+ 2.95	0.10–5.80	0.043
Post-intervention time	0.00	−0.24–0.24	1.00	−0.09	−0.33–0.15	0.45

## Discussion

This study examined the impact of implementing a structured physician shift-based work system in a Japanese emergency and critical care center. The introduction of the shift system was not associated with discernible changes in in-hospital mortality. In contrast, findings related to LOS differed by analytical approach. Crude pre–post comparisons demonstrated a shorter LOS in the post-shift period. However, ITS analysis incorporating adjustment for temporal changes in patient severity and diagnostic case-mix identified a significant immediate increase in LOS at the time of implementation, without evidence of a sustained change in the post-intervention trend.

The absence of a significant change in in-hospital mortality following the implementation of a shift-based physician work system is consistent with previous studies reporting no adverse impact of duty hour reforms or shift scheduling on patient survival in acute care settings [2, 3]. For example, studies evaluating resident duty hour restrictions in the United States and Europe found no increase in mortality or serious adverse events following reform implementation [2, 7]. While concerns have been raised about decreased continuity of care and increased handoffs under shift systems [8, 9], structured communication protocols and team-based care models may help mitigate these risks [10]. In recent years, global attention has shifted toward improving physician wellness and reducing excessive work hours, with many healthcare systems transitioning toward structured shift-based staffing models [7, 11]. Our findings are in line with this literature, suggesting that such structural changes, when implemented thoughtfully, do not necessarily correspond to deterioration in critical patient outcomes such as in-hospital mortality.

On the other hand, an increase in LOS was observed at the time of shift system implementation. Although crude comparisons suggested a shorter LOS in the post-shift group, analyses that accounted for temporal trends using ITS methods demonstrated an immediate level increase in LOS around the implementation period. Previous reports have suggested that the introduction of new staffing models may disrupt established workflows, particularly in high-intensity settings such as intensive care units [12, 13]. Potential contributors to such changes include delays in clinical decision-making, reduced familiarity with individual patients due to increased handoffs, and adaptation to newly introduced workflows [8–10]. Even if transient, such disruptions may place additional burdens on hospital resources and bed availability. Importantly, no sustained change in the post-intervention LOS trend was observed, suggesting that the observed increase was temporally limited to the implementation phase. This finding underscores the importance of careful transitional planning when introducing shift-based physician work systems, particularly in high-acuity environments such as emergency and critical care units [14, 15]. Accordingly, implementation strategies should incorporate transitional support mechanisms, including structured onboarding, standardized handover protocols, simulation-based training, and robust interprofessional communication frameworks, to promote operational continuity and patient flow efficiency. At our institution, structured orientation programs for newly assigned staff and residents, as well as regular multidisciplinary conferences, were introduced alongside the shift system, which may have contributed to stabilization of LOS following the initial implementation period.

Importantly, this study applies an ITS approach to evaluate the clinical impact of a shift-based physician work system in a real-world Japanese emergency and critical care setting. While previous studies have examined duty hour reforms or shift scheduling primarily in Western healthcare systems [1–3], evidence from Japan—where physician workforce structures, training pathways, and hospital operations differ substantially—has remained limited. In addition, the intervention evaluated in this study involved a comprehensive restructuring of physician scheduling across all levels of clinical practice, encompassing both attending physicians and resident trainees, rather than a partial modification restricted to resident duty hours alone. By analyzing more than seven years of longitudinal data and over 5,000 admissions, this study contributes to the existing literature by examining both immediate level changes and longer-term trends in key clinical outcomes using a time series framework [16–18]. The ITS design enabled separation of secular trends from changes temporally associated with implementation, and supplementary analyses incorporating monthly mean APACHE II scores and diagnostic category distributions helped to contextualize observed patterns in relation to patient severity and diagnostic composition over time. Collectively, these methodological features strengthen the interpretability of the findings and provide empirically grounded insights relevant to ongoing discussions of physician work style reform in high-acuity care environments. In addition to patient-centered outcomes, future studies should also examine workforce-related measures such as physician burnout, job satisfaction, and staff retention, which are central to the goals of work style reform.

This study has several limitations. First, it was conducted at a single academic emergency and critical care center in Japan, which may limit the generalizability of the findings to institutions with different staffing models, patient populations, or healthcare systems. Second, although elective admissions, cardiopulmonary arrest cases, and patients admitted during the COVID-19 pandemic period were excluded to reduce major sources of confounding, residual confounding from unmeasured factors—such as seasonal variation, secular changes in clinical practice, or subtle shifts in case mix—cannot be fully excluded. Third, the ITS analysis was based on aggregated monthly data rather than patient-level observations, which limited the granularity of risk adjustment and reduced the number of effective data points. While ITS methods account for underlying temporal trends, they do not fully eliminate the influence of time-varying confounders. We attempted to address this limitation by incorporating monthly mean APACHE II scores and diagnostic category distributions; however, residual confounding may remain. Although the shift-based work system was formally implemented on October 1, 2023, a short transitional or phased implementation period in routine practice cannot be excluded. Excluding months around the intervention would have substantially reduced the number of post-intervention observations and limited statistical power. Therefore, we modeled the intervention as a single time point, which is a commonly accepted approach in interrupted time series analyses of health system interventions. Fourth, objective working-hour data were only available after implementation because electronic time-stamping was not mandatory beforehand, precluding direct before–after comparisons. Fifth, the study focused on two outcomes—in-hospital mortality and ICU length of stay—which may not fully capture other clinically relevant or operational consequences of shift-based physician scheduling, such as ICU readmissions, ventilator-free days, handoff quality, or measures of care continuity. Sixth, although compliance with the nationally mandated overtime limits was complete during the study period, we were unable to quantitatively assess adherence to other components of the reform, including the quality of handovers, workflow adaptation, or team communication processes. Finally, this analysis primarily reflects the early phase following implementation of the shift-based system. Longer-term effects on patient outcomes, care processes, physician well-being, and staff retention warrant further investigation with extended follow-up. With longer post-intervention follow-up, future studies may be better positioned to model phased or transitional implementation periods and to more precisely characterize temporal patterns of change.

## Conclusion

In this single-center ITS study conducted in a Japanese emergency and critical care center, implementation of a structured physician shift-based work system was not associated with an increase in in-hospital mortality. A transient increase in ICU length of stay was observed at the time of implementation, without evidence of sustained post-intervention trend changes. These findings describe temporal changes in selected clinical outcomes following system implementation but should be interpreted with caution given the potential for residual confounding and the limitations inherent to observational time series analyses.

## Supplementary Information

Below is the link to the electronic supplementary material.

Supplementary Material 1: Suppl. Fig.1 Monthly proportion of patients with APACHE II score ≥20 during the study period. The line graph shows the monthly proportion of patients with APACHE II score ≥20 from November 2017 to December 2024

Supplementary Material 2

## Data Availability

The datasets supporting the conclusions of this article will be provided based on reasonable request to the corresponding author.

## Abbreviations

ACGME: Accreditation Council for Graduate Medical Education
APACHE: Acute physiology and chronic health evaluation
CRRT: Continuous renal replacement therapy
ED: Emergency department
GCS: Glasgow Coma Scale
ICU: Intensive care unit
IQR: Interquartile range
ITS: Interrupted time series
LOS: Length of stay
SD: Standard deviation
