# Supplementary figures and images for: Impact of physician shift work implementation on mortality and length of stay in an emergency and critical care center: an interrupted time series analysis
Source: BMC Health Serv Res. 2026 Feb 18;26:397. doi: 10.1186/s12913-026-14226-6 (PMC13020193; doi:10.1186/s12913-026-14226-6)

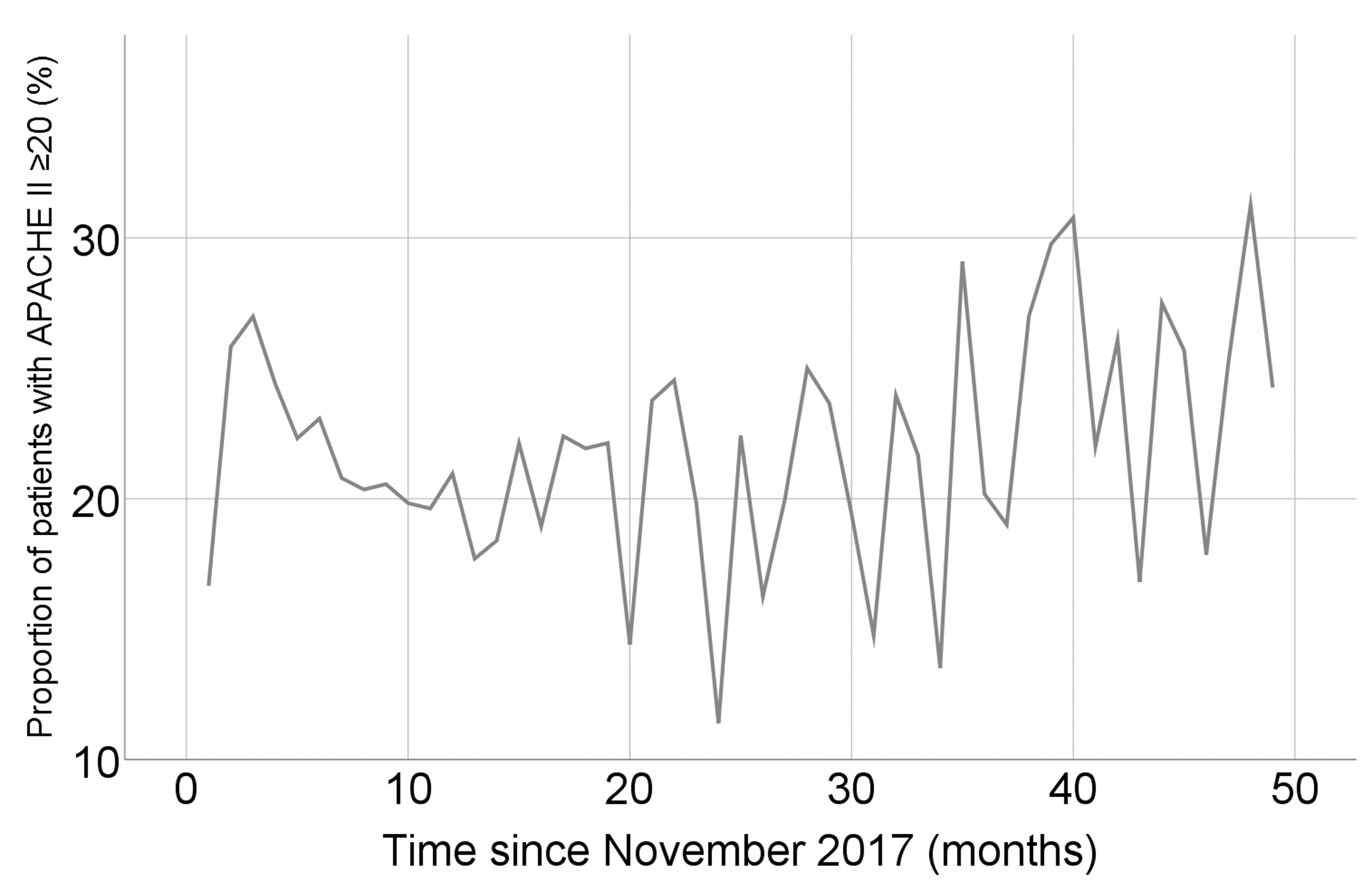

Supplement: Supplementary file 1 — Supplementary Material 1: Suppl. Fig.1 Monthly proportion of patients with APACHE II score ≥20 during the study period. The line graph shows the monthly proportion of patients with APACHE II score ≥20 from November 2017 to December 2024 [file 12913_2026_14226_MOESM1_ESM.tif]
