# Supplementary material for: Impact of physician shift work implementation on mortality and length of stay in an emergency and critical care center: an interrupted time series analysis
Source: BMC Health Serv Res. 2026 Feb 18;26:397. doi: 10.1186/s12913-026-14226-6 (PMC13020193; doi:10.1186/s12913-026-14226-6)
Supplement: Supplementary file 2 — Supplementary Material 2 [file 12913_2026_14226_MOESM2_ESM.docx]

**Supplementary Table 1** Monthly median physician working hours after implementation of the shift-based physician work system, stratified by postgraduate year

| Postgraduate year | Monthly working hours, median (IQR), hours per month | |
| --- | --- | --- |
| 1–2 | 8.2 | (6.8–9.5) |
| 3–5 | 43.0 | (28.1–65.1) |
| 6–36 | 51.0 | (35.0–70.0) |

**Supplementary Table 2** Monthly trend analysis of high-severity case proportion (APACHE II score ≥20)

| Variable | *n* (months) | Kendall’s *τ* | *p* |
| --- | --- | --- | --- |
| APACHE II score ≥20 (%) | 49 | 0.15 | 0.13 |
